# A Changing Gastric Environment Leads to Adaptation of Lipopolysaccharide Variants in *Helicobacter pylori* Populations during Colonization

**DOI:** 10.1371/journal.pone.0005885

**Published:** 2009-06-11

**Authors:** Anna Skoglund, Helene Kling Bäckhed, Christina Nilsson, Britta Björkholm, Staffan Normark, Lars Engstrand

**Affiliations:** 1 Department of Microbiology, Tumor and Cell Biology, Karolinska Institute, Stockholm, Sweden; 2 Swedish Institute for Infectious Disease Control, Solna, Sweden; University of Hyderabad, India

## Abstract

The human gastric pathogen *Helicobacter pylori* colonizes the stomachs of half of the human population, and causes development of peptic ulcer disease and gastric adenocarcinoma. *H. pylori*-associated chronic atrophic gastritis (ChAG) with loss of the acid-producing parietal cells, is correlated with an increased risk for development of gastric adenocarinoma. The majority of *H. pylori* isolates produce lipopolysaccharides (LPS) decorated with human-related Lewis epitopes, which have been shown to phase-vary in response to different environmental conditions. We have characterized the adaptations of *H. pylori* LPS and Lewis antigen expression to varying gastric conditions; in *H. pylori* isolates from mice with low or high gastric pH, respectively; in 482 clinical isolates from healthy individuals and from individuals with ChAG obtained at two time points with a four-year interval between endoscopies; and finally in isolates grown at different pH *in vitro*. Here we show that the gastric environment can contribute to a switch in Lewis phenotype in the two experimental mouse models. The clinical isolates from different human individuals showed that intra-individual isolates varied in Lewis antigen expression although the LPS diversity was relatively stable within each individual over time. Moreover, the isolates demonstrated considerable diversity in the levels of glycosylation and in the sizes of fucosylated O-antigen chains both within and between individuals. Thus our data suggest that different LPS variants exist in the colonizing *H. pylori* population, which can adapt to changes in the gastric environment and provide a means to regulate the inflammatory response of the host during disease progression.

## Introduction

*Helicobacter pylori* colonization leads to gastritis in virtually all infected hosts, and a subset progresses to peptic ulcer, gastric adenocarcinoma or MALT lymphoma [1]. Chronic atrophic gastritis (ChAG), which is considered to be a precancerous state, is associated with loss of acid-producing parietal cells (and hence an increase in gastric pH) and pepsinogen-producing zymogenic cells [2]. During disease progression, the gastric environment changes and the infecting *H. pylori* strains must adapt to persist in a gastric habitat with increased pH, a new gastric cell composition and invasion of intestinal microbes.

A combination of microbial, host and environmental factors contribute to gastric disease development. *H. pylori* encodes several virulence factors, the most well-described being the *cag* pathogenicity island (PAI), which encodes a type IV secretion system and the effector protein CagA, and the vacuolating cytotoxin VacA, which induces morphogenic changes of the host cell [3]–[5]. Additionally, *H. pylori* possesses the ability to phase-vary genes encoding outer membrane proteins as well as genes involved in lipopolysaccharide (LPS) biosynthesis, which enables adaptation to varying gastric conditions [6]–[9].

LPS is the major component of the cell wall in Gram-negative bacteria and is estimated to occupy 75% of the cell surface [10]. The LPS molecule is composed of a lipid A part, a core oligosaccharide unit and a variable O-antigen chain. The O-antigen chain of *H. pylori* is uniquely decorated with host-related Lewis antigens, carbohydrates that are also expressed by the gastric epithelium in humans. These structures have been suggested to be important for gastric colonization, adhesion and immune evasion through molecular mimicry where the Lewis antigens provide a “camouflage” for the bacteria in order to escape the host immune response [11]. Moreover, the phase-variable expression of Lewis antigens allows *H. pylori* to modulate the host T-helper cell immune response through interactions with DC-SIGN on dendritic cells [12], which might facilitate persistent colonization.

Lewis x (Le^x^) and Lewis y (Le^y^), the dominant Lewis antigens in *H. pylori* LPS, are expressed by 80–90% of clinical isolates [13]–[16]. Other related antigens, such as Lewis a (Le^a^), Lewis b (Le^b^), Lewis c (Le^c^), sialyl-Le^x^ and H-antigens, may also be expressed by *H. pylori*, although at lower frequencies [17]–[19]. Le^x^ and Le^y^ are synthesized by the addition of fucose residues to *N*-acetyl-β-lactosamine (LacNAc) units in the O-antigen chain, which is typically glycosylated with internal Le^x^ units and either Le^x^ or Le^y^ at the terminal position [9]. Lewis antigen biosynthesis requires the action of three fucosyltransferases, FutA, FutB and FutC. FutA and FutB may have either α1,3- and/or α1,4-fucosyltransferase activity for Le^x^ or Le^a^ antigen synthesis, respectively. In a second step, FutC with α1,2-fucosyltransferase activity adds an additional fucose which results in Le^y^ or Le^b^ antigen synthesis [11], [20]. The expression of all three fucosyltransferases is regulated by a slipped-strand mispairing mechanism due to stretches of C-residues in the 5′end of the *futA*, *futB* and *futC* genes, which yields variants with different LPS phenotypes [6], [21].

Moreover, FutA and FutB each contain a variable heptad-repeat region that functions as a molecular ruler. The number of repeated heptads directs which sizes of O-antigen polymers to become fucosylated [22]. Depending on external conditions, such as the surrounding environment, there seem to be a selection for fucosylation of diverse sizes of O-antigen chains [23]. Thus, *H. pylori* LPS phenotypes can vary both between different strains [24] as well as within a single strain [6], [22], [23]. Interestingly, expression of Le^x^ and Le^y^ has been shown to be influenced by pH and iron levels *in vitro* [25], [26] and vary in different gastric regions and within a single host [22]. Therefore, Lewis antigens play an important role in the interaction with the host, which can contribute to disease development.

The aim of this study was to investigate how Lewis antigen expression adapts to varying gastric and environmental conditions seen in normal and atrophic individuals with low and high gastric pH, respectively. We analyzed the LPS of *H. pylori* strain HPAG1, obtained from an individual with ChAG, after one year of colonization in a germ-free human ChAG mouse model (*tox*176 mice) with neutral gastric pH, and in non-transgenic FVB/N mice with low gastric pH. We also examined the Lewis antigen diversity over time in a unique strain material of 482 clinical *H. pylori* single-colony isolates obtained from 17 individuals, including healthy controls and individuals with ChAG or gastric adenocarcinoma, that were endoscoped twice with a four-year interval. Finally, the effect of pH on LPS and Lewis antigen expression was determined by culturing a panel of *H. pylori* isolates *in vitro* at pH 5 and 7 prior to LPS isolation and Lewis antigen phenotyping.

## Results

### Effects on *H. pylori* LPS and Lewis antigen expression by colonization in different murine gastric environments

**Figure 1:**
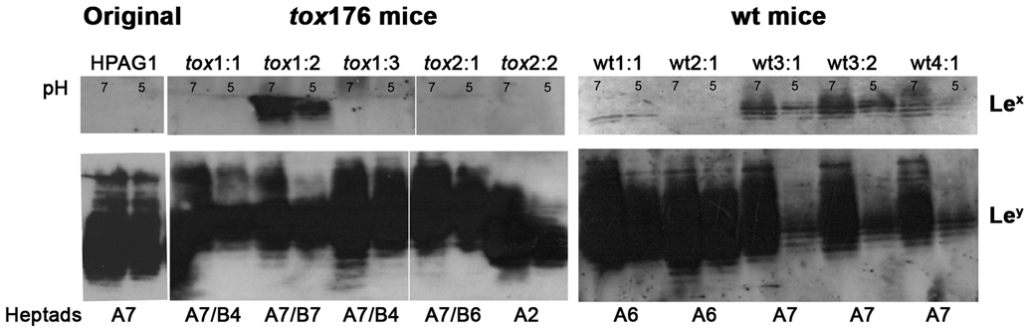
Lewis phenotypes of HPAG1 isolates after a one-year colonization in germ-free *tox*176 and wildtype mice. *H. pylori* strain HPAG1 was used for a one-year colonization of germ-free *tox*176 and non-transgenic (wildtype) mice with high and low gastric pH, respectively. LPS was extracted from five single-colony re-isolates from *tox*176 mice (n = 2) and five single-colony re-isolates from wildtype mice (n = 4) grown *in vitro* at both pH 7 and pH 5. Immunoblot analysis with antibodies detecting Le^x^ and Le^y^ showed that isolates from wildtype mice had switched Lewis phenotype to simultaneous Le^x^ and Le^y^ expression, as compared to the inoculating HPAG1 strain and the *tox*176-passaged HPAG1 isolates. Some isolates (wt3∶1, wt3∶2 and wt4∶1) appeared to express O-antigen chains that were less fucosylated when grown *in vitro* at pH 5 as compared to growth at pH 7. DNA sequencing showed that; FutA was “on” in all isolates; FutB was “on” in four *tox*176-passaged isolates (*tox1*:1-1:3 and *tox*2:1); and the number of heptad-repeats varied in active FutA and FutB enzymes, which is indicated below the lanes.

To study the effect of different gastric environments and gastric pH on Lewis antigen expression, we analyzed the LPS of five HPAG1 single-colony re-isolates obtained after one year of colonization of parietal cell-deficient *tox*176 mice (n = 2) and five HPAG1 single- colony re-isolates from wildtype mice (n = 4) [27]. Immunoblotting showed that the infecting HPAG1 strain expressed exclusively Le^y^-glycosylated O-antigen chains (Figure 1). However, four of the five isolates from the wildtype mice had switched Lewis antigen phenotype and expressed O-antigen chains that were both Le^x^- and Le^y^-glycosylated (Figure 1), while four of five isolates from the *tox*176 mice expressed O-antigen chains that were exclusively Le^y^-glycosylated (like the infecting HPAG1 strain). The banding patterns, representing varying O-antigen chain lengths, showed that there was a tendency towards more diversity in sizes of Le^y^-glycosylated O-antigen chains among the strains from the *tox*176 mice than among the strains from the wildtype mice. This might be a reflection of the different numbers of heptad-repeats in the expressed fucosyltransferases (see below).

The original HPAG1 isolate, used for mouse infection, produced Le^y^, and no Le^x^, arguing that the *futC* gene must be expressed. Sequence analyses further revealed that this isolate expressed FutA with seven heptad-repeats, whereas the *futB* gene was out of frame (Table 1). All mouse-passaged isolates retained *futA* in-frame, however, they varied in the number of heptad-repeats. The *futB* gene, however, remained out of frame for all isolates passaged in wildtype mice but in-frame for four of the five *tox*176-passaged isolates (Figure 1 and Table 1). The number of heptad-repeats among the four *tox*176-passaged isolates with expressed *futB* was either four, six or seven. Interestingly, the isolates with non-expressed *futB* had also switched from five heptads in the inoculating HPAG1 strain, to six or eight heptad-repeats (Table 1). PCR analyses of the mouse-passaged isolates confirmed that all isolates were *cagA* positive and carried a complete cag PAI, like the HPAG1 strain (data not shown).

**Table 1:** Sequencing results of *futA* and *futB* of HPAG1 isolates after a one-year colonization of germ-free *tox*176 and wildtype (wt) mice.

*H. pylori* isolates	*futA*		*futB*	
	C-repeat	Heptads	C-repeat	Heptads
HPAG1*	5	7	12	5
*tox*1:1	5	7	11	4
*tox*1:2	5	7	11	7
*tox*1:3	5	7	11	4
*tox*2:1	5	7	14	6
*tox*2:2	5	2	13	6
wt1∶1	5	6	12	8
wt2∶1	5	6	14	6
wt3∶1	5	7	14	6
wt3∶2	5	7	13	6
wt4∶1	5	7	13	6

* Original inoculating strain for the colonization.

### Effects on *H. pylori* LPS and Lewis antigen expression in mouse-passaged isolates after growth at different pH

**Figure 2:**
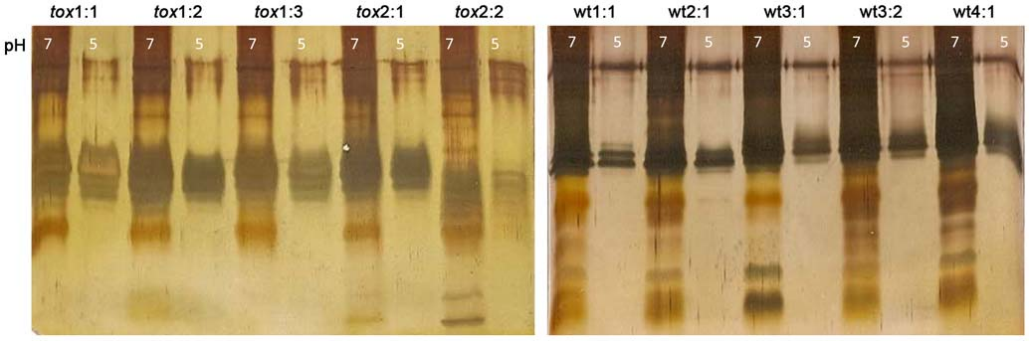
LPS profiles of HPAG1 isolates after a one-year colonization in germ-free wildtype and *tox*176 mice. Silver staining of LPS from HPAG1 single-colony re-isolates after a one year-colonization of germ-free *tox*176 (tox) and non-transgenic wildtype (wt) mice grown *in vitro* at pH 7 and pH 5. The analyses revealed that LPS from isolates grown at pH 7 displayed significantly higher levels of LPS expression than the isolates grown at pH 5, which demonstrated high-and low-molecular weight structures that were only weakly expressed or completely absent.

The lack of parietal cells in the *tox*176 mice results in a gastric environment with elevated pH for *H. pylori* as compared to colonization in wildtype mice. To see if these two *in vivo* growth conditions had pH-regulated effects on LPS and Lewis antigen expression, we first compared silver stained LPS profiles of HPAG1 re-isolates after *in vitro* growth at pH 7 and pH 5, respectively. As seen in Figure 2, all isolates grown at pH 5 displayed significantly lower levels of LPS expression and demonstrated an absence of, or very weak expression, of many of the high-and low-molecular weight structures present in the LPS profiles of isolates grown at pH 7. Immunoblotting revealed that the Lewis phenotypes *per se* were unaffected by *in vitro* growth at different pH (Figure 1). Nevertheless, three of the five isolates passaged in wildtype mice (wt3∶1, wt3∶2, and wt4∶1) expressed considerably less Lewis antigens at pH 5 as compared to pH 7. Interestingly, this phenotype was not as apparent for the original HPAG1 isolate and for the five isolates retrieved from *tox*176 mice, suggesting that *in vivo* growth at the higher pH prevailing in an atrophic gastric environment may affect pH regulation of *H. pylori* Lewis antigen expression.

### Effects on *H. pylori* Lewis antigen expression in clinical isolates from different human gastric environments

To examine whether Lewis antigen expression was changed over time as an effect of atrophy development (e.g. an effect of elevated gastric pH), we analyzed the LPS of 482 clinical *H. pylori* isolates from corpus biopsies of 17 individuals obtained from two endoscopies with four years apart. First we analyzed the levels of pepsinogen I and II in serum collected at the first endoscopy to verify the histological diagnosis of atrophic gastritis. The histological classification was confirmed in all cases and the subjects were categorized as normal or atrophic. Isolates from individuals Kx201 and Kx345, who were classified as normal at the first time point but had developed atrophy at the four-year follow-up, as well as isolates from Kx438, who was diagnosed with atrophy at the first time point and gastric adenocarcinoma at the second time point, were considered “atrophy-associated isolates” at both time points.

**Figure 3:**
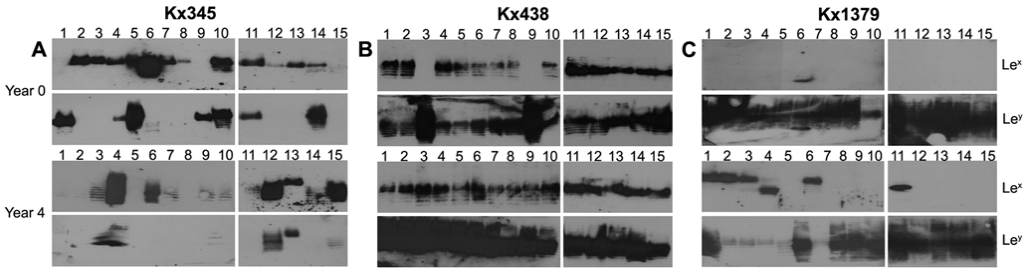
Lewis antigen expression in clinical *H. pylori* isolates shows large intra-individual diversity. Western blots detecting Le^x^ and Le^y^ antigens. Fifteen single-colony isolates were analyzed from each individual and time point. (A) High variability of Lewis antigen pattern was usually observed among intra-individual isolates, e.g. in single-colony isolates from Kx345 where large variation in sizes of Le^x^ and Le^y^ fucosylated O-antigen chains were detected. Here all the four different Lewis antigen phenotypes are represented: those expressing both Le^x^ and Le^y^, isolates that only expressed Le^x^ or Le^y^ and strains that lacked expression of both these Lewis antigens. (B) Kx438 isolates simultaneously expressed both Le^x^ and Le^y^ at both time points, except two isolates at year 0 (isolate 3 and 9) that only expressed Le^y^, but with comparably higher intensities. (C) The Kx1379 isolates show how the amount of Lewis antigen expression can vary in expression intensities, e.g. at year 4, where low amounts of Le^y^ were expressed in isolates 2, 3, 4, 5 and 7 as compared to isolates 1, 6 and 8–15, where more pronounced Le^y^ expression was observed.

To assess the variability of LPS phenotypes within an individual's *H. pylori* population, we compared banding patterns on the immunoblots, which represent diverse sizes of O-antigen chains that are Le^x^- and/or Le^y^-glycosylated. Fifteen single-colony isolates from 17 individuals were examined, of which three representative examples are given in Figure 3, and the remaining isolates in Figure S1. We were able to identify intra-strain diversity of Lewis epitopes in the same microenvironment, i.e. the 15 single-colony isolates from the same biopsy presented diverse Lewis antigen profiles with immunoblotting (Figures 3A-C and Figure S1). Both expression levels, pattern of Lewis glycosylation and the sizes of O-antigen chains that were fucosylated, varied in isolates obtained from the same individual. This indicates an advantage of maintaining a high phenotypic diversity of the LPS molecule within the bacterial community. Often, Le^x^-glycosylated O-antigen chains were restricted to only a few sizes, while the Le^y^ antigen was present on O-antigen chains of several sizes. This phenomenon was observed in isolates from both normal and atrophic individuals, and typically when occurring, found at both time points. Thus the diversification seemed to be sustained within isolates from the same individual. However, no correlation in expression intensity was found over time or in association with disease development.

**Figure 4:**
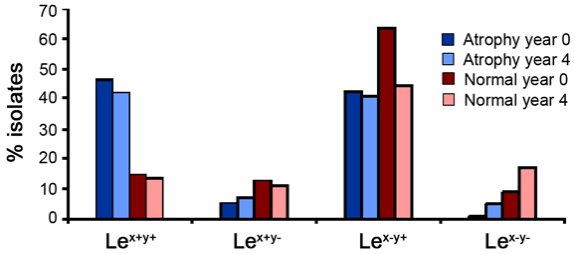
Lewis antigen expression in clinical *H. pylori* isolates from individuals with atrophy and normal controls. The average percent of Lewis phenotypes (Le^x+y+^, Le^x+y−^, Le^x−y+^ and Le^x−y−^) of single-colony isolates from each individual, categorized as normal or atrophic are shown. Dark blue and light blue-coloured bars: average percent of isolates obtained from atrophic individuals year 0 and year 4, respectively. Dark red and light red-coloured bars: average percent of isolates obtained from normal individuals year 0 and year 4, respectively. Lewis expression was stable over time within individuals and Lewis phenotype frequencies were significantly correlated between year 0 and year 4 (Spearman's rank correlation *P*<0.05).

Isolates from each individual were divided into four phenotype groups depending on the Lewis antigens expressed; (1) isolates expressing both Le^x^ and Le^y^, (2) isolates expressing only Le^x^, (3) isolates expressing only Le^y^, and (4) isolates with expression of neither Le^x^ nor Le^y^ (Table 2 and Figure 4). The proportions of isolates in each phenotype group were remarkably stable over time within individuals and phenotype frequencies were significantly correlated between year 0 and year 4 (Figure 4; Spearman's rank correlation *P*<0.05). This was true for normal as well as atrophy individuals. Notably, we observed a non-significant trend towards higher frequencies of simultaneous expression of Le^x^ and Le^y^ among *H. pylori* isolates from individuals with atrophy as compared to normal individuals (T-test, *P* = 0.08). The most common LPS phenotype was Le^y^, either alone, or in combination with Le^x^ (50% and 32%, respectively). In contrast, least common was Le^x^ expression exclusively, which was found in 8.5% of the 482 clinical isolates (Table 2, Figures 3 and S1), suggesting a selection for maintaining *futC* in- frame.

**Table 2:** Lewis phenotypes and *cag* status of clinical *H. pylori* isolates obtained from the Kalixanda study.

Individual	Histopathology at year 0 and 4	Le^x+y+^	Le^x+y−^	Le^x−y+^	Le^x−y−^	*cag* status
**Kx438**	Moderate atrophy	13	0	2	0	+
	Gastric adenocarcinoma	15	0	0	0	+
**Kx201**	Normal	8	0	7	0	+
	Mild atrophy	11	1	3	0	+
**Kx345**	Normal	6	6	2	1	+
	High grade atrophy	4	6	0	5	+
**Kx439**	Mild atrophy	4	0	11	0	+
	High grade atrophy	ND	ND	ND	ND	ND
**Kx533**	Moderate atrophy	4	0	11	0	+
	High grade atrophy	2	0	13	0	+
**Kx1039**	Mild atrophy	8	0	7	0	+
	High grade atrophy	14	0	1	0	+
**Kx1167**	Moderate atrophy	3^1^	0^1^	0^1^	0^1^	+^1^
	High grade atrophy	2	0	13	0	+
**Kx1172**	Moderate atrophy	4	0	11	0	+
	High grade atrophy	2	0	13	0	+
**Kx208**	Normal	2	1	7	5	+
	Normal	0	0	7	8	+
**Kx239**	Normal	0	0	15	0	+/− 3
	Normal	0	0	15	0	+
**Kx364**	Normal	11	0	4	0	-4
	Normal	13	2	0	0	-4
**Kx491**	Normal	0	15	0	0	-
	Normal	0	15	0	0	-
**Kx573**	Normal	5	1	4	5	+
	Normal	4	0	9	2	+
**Kx595**	Normal	0	0	15	0	-
	Normal	9	0	6	0	-
**Kx1259**	Normal	0	0	13	2	+
	Normal	0	0	2	13	+
**Kx1353**	Normal	1^2^	0^2^	13^2^	0^2^	+
	Normal	3	0	12	0	+
**Kx1379**	Normal	1	0	14	0	+
	Normal	6	0	9	0	+
**Total**		**155**	**47**	**239**	**41**	
		**(32%)**	**(9.8%)**	**(50%)**	**(8.5%)**	

1 3 isolates analyzed.

2 14 isolates analyzed.

3 80% positive for *cagA* and 20% positive for *cag* PAI empty site PCR in this individual.

4 All isolates were negative for both *cagA* and *cag* PAI empty site PCR:s, indicating that part of the *cag* PAI may still be present in these isolates, although the *cagA* gene is deleted.

**Figure 5:**
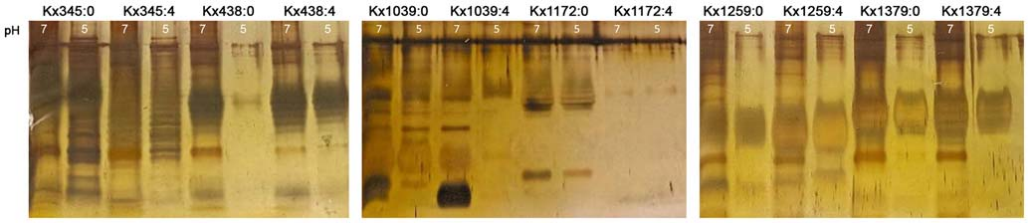
LPS profiles of clinical *H. pylori* isolates grown *in vitro* at pH 7 and pH 5. Silver staining of LPS from clinical *H. pylori* single-colony isolates grown *in vitro* at pH 7 and pH 5, revealed that some of the isolates grown at pH 5 expressed less LPS than isolates grown at pH 7, e.g. isolates Kx1259 and Kx1379 obtained from normal individuals. These isolates presented LPS that lack some of the high- and low-molecular structures in the pH 5 samples as compared to isolates grown at pH 7. The atrophy-associated isolates Kx345, Kx438, Kx1039 and Kx1172 presented similar LPS profiles after growth at pH 7 and pH 5, except Kx438:0 and Kx1039:4, which showed weaker LPS expression at pH 5 as compared to pH 7.

One single-colony isolate from six individuals (Kx345, Kx438, Kx1039, Kx1172, Kx1259 and Kx1379) at both time points (n = 12) were grown *in vitro* at pH 7 and pH 5 prior to LPS isolation. Silver staining showed that isolates from normal individuals carried LPS that lacked some of the high- and low-molecular structures after growth at pH 5 as compared to isolates grown at pH 7 (Figure 5). The atrophy-associated isolates Kx345:0, Kx345:4, Kx438:4, Kx1039:0, Kx1172:0 and Kx1172:4 presented similar LPS profiles after growth at pH 7 and pH 5, whereas Kx438:0 and Kx1039:4 showed weaker LPS expression at pH 5 as compared to pH 7. Hence, pH regulation of LPS expression seen in isolates from a healthy gastric environment is frequently lost in atrophy-associated isolates (Figure 5). Immunoblotting showed that culturing at different pH *in vitro* did not have an effect on Lewis antigen expression *per se*. The expression levels of Lewis antigens at pH 7 and pH 5 differed slightly, e.g. in isolates Kx345:4, Kx438:0 and Kx1379:4, which showed reduced expression of Le^y^ at pH 5 as compared to pH 7 (Figure S2).

### Genetic analyses of clinical *H. pylori* isolates

To investigate the relatedness of the *H. pylori* isolates in each individual, genomic fingerprinting, using RAPD-PCR, was performed on randomly chosen clinical isolates. Isolates originating from the same individual produced related RAPD-PCR patterns, although distinct from isolates from other individuals, which indicate that most individuals were colonized by *H. pylori* isolates of the same strain origin. However, subtle differences in the banding pattern appeared, indicating small genetic changes of *H. pylori* strains during the course of infection (data not shown).

Presence of *cagA* has earlier been correlated with Lewis antigen expression [15], [28]. Therefore, the *cag* status of the clinical isolates was analyzed by PCR using DNA isolated from ten colonies at both time points (Table 2). Primers detecting both *cagA* and flanking regions of the *cag* PAI (*cag* PAI empty site PCR) were used for the analyses. Remarkably, all atrophy-associated isolates (n = 143) were *cagA* positive and *cag* PAI empty site negative, indicating presence of a complete *cag* PAI (Table 2). In the normal group (n = 180), two out of nine individuals (Kx491 and Kx595) were colonized with *H. pylori* that were *cagA* negative and *cag* PAI empty site positive, indicating deletion of the entire *cag* PAI. Another individual (Kx364) carried *H. pylori* isolates that were both *cagA* negative and cag PAI empty site negative, indicating that part of the *cag* PAI may still be present in these isolates, although the *cagA* gene is deleted. Finally, isolates from yet another individual (Kx239) were both *cagA* positive (80%) and *cag* PAI empty site positive (20%), suggesting that the *cag* PAI has been excluded from some of these isolates, as described elsewhere [29], [30]. Thus, of the 180 isolates in the normal group, 118 isolates (66%) carried a complete *cag* locus. We found, however, no significant correlation between presence of *cagA* and Lewis antigen expression among the isolates.

## Discussion

Our data show that different gastric environments with distinct pH affect Lewis antigen expression in *H. pylori* LPS during colonization in mice. Moreover, although bacterial sub-populations expressing different Lewis antigens co-exist within human stomachs, the frequencies of Lewis phenotypes are stable during a persistent infection. The clinical isolates showed that the on/off Lewis phenotype pattern was relatively stable over time in single individuals and was not significantly changed upon transition from a normal to an atrophic gastric mucosa. However, the observed intra-individual strain diversity, with regard to levels of Lewis antigen expression and sizes of Lewis glycosylated O-antigen chains, suggests an advantage for altering these characteristics rather than the Lewis antigen repertoire *per se* in a bacterial population. As previously described [13], [31], we also observed that disease-associated isolates showed a higher tendency to present LPS that were both Le^x^- and Le^y^-glycosylated, while the LPS of isolates obtained from normal mucosa generally presented either Le^x^ or Le^y^ exclusively, or none. However, the difference was not large enough to be statistically significant.

A previous *in vitro* study has described pH as an environmental regulator of Lewis antigen phase variation [25]. In order to investigate this in an *in vivo* setting, we colonized two germ-free mouse models with low or high gastric pH, mimicking normal gastric conditions and ChAG, respectively, with *H. pylori* strain HPAG1 for one year. After the colonization, isolates from the wildtype mice demonstrated a switch from solely Le^y^ glycosylation to both Le^x^ and Le^y^ glycosylation, while the *tox*176-passaged isolates remained Le^y^-glycosylated like the inoculating strain. Thus, Le^x^ and Le^y^ were expressed in stomachs with a lower gastric pH (pH 2–5) and Le^y^ in stomachs with at a neutral pH. The switch in Lewis antigen expression suggests that different gastric environments, and possibly gastric pH, can influence the fucosylation of LPS. Interestingly, all switches in fucosyltransferase activity observed in the isolates resulted in an on-switch rather than turning the Lewis antigen expression off.

The ablation of parietal cells in the *tox*176 mice is associated with loss of the pepsinogen-producing zymogenic cells and an increased proliferation of multi-potent gastric stem cells [32]. The variable Lewis antigen expression is likely an effect of long-term colonization in two gastric habitats with different pH and different cell composition, which is determined by the presence or absence of functional parietal cells. Consequently, the colonizing strains can vary their Lewis antigen expression as a means of adapting to the different gastric environments. Colonization studies in Rhesus monkeys have demonstrated that the Lewis antigen expression of *H. pylori* was adapted to match the corresponding Lewis-phenotype of the host [33]. A similar correlation has been described in humans [34], however, this has been disputed by others [13], [35].

Our investigation of Lewis antigen expression in clinical *H. pylori* isolates obtained from individuals in the Kalixanda study, made it possible to compare isolates from individuals who exhibit a normal gastric mucosa at both time points with isolates from individuals during atrophy development. The 15 single-colony isolates obtained from each of 17 individuals at two different time points displayed high intra-individual variability, indicating that the bacterial population in each individual consists of a mix of different LPS phenotypes. Even though there is a considerable intra-individual variability, it appears that there is a selection for maintaining the fucosyltransferase genes on so that both Le^x^ and/or Le^y^ are expressed. Overall, the majority of isolates, 91%, expressed both Le^x^ and Le^y^ simultaneously or alone. Among the atrophy-associated isolates, 97% presented Le^x^- and/or Le^y^-glycosylated LPS while among the isolates from normal individuals, this proportion was 87%. Thus, there were very few isolates, 41 out of 482 single-colony isolates (8.5%) that did not express Le^x^ or Le^y^, which are the most commonly found Lewis antigens in *H. pylori* LPS. The majority of these isolates were obtained from normal individuals without symptoms of disease, which has also described by others [13], [31].

Moreover, we showed that there is no change in frequency of Lewis antigen expression within the individuals over the four-year period in neither normal nor atrophic individuals, suggesting that there is no selection in the gastric environment that promotes such a change. Thus, the reduced acid output associated with gastric atrophy did not appear to affect the Lewis phenotypes in humans. Our data suggest that the Lewis antigen repertoire of strains infecting normal individuals is not the same as for strains infecting individuals with atrophy (Table 2, Figures 3 and S1); however whether the Lewis expression itself contributes to disease remains to be further investigated. Previous studies have reported that Lewis antigen expression is altered in hosts during a persistent infection. However these studies did not analyze multiple isolates from each time point, rather one solely single-colony isolate [36], or the whole bacterial population [37] was examined, and the time-interval was seven to ten years, or one to two months, respectively.

Here, we have analyzed clinical *H. pylori* isolates from individuals at two different time points which represent a snapshot of how the Lewis antigen evolves in the infected host. Since *H. pylori* colonization is established during early childhood and persists throughout the lifetime of the host, it is likely that the host environment initially selects for certain LPS phenotypes although new variants may evolve during the course of infection. Since transmission of *H. pylori* occurs within families, and predominantly between mother-child, it would be interesting to investigate LPS phenotypes of family members to assess the adaptation of *H. pylori* at an early stage to elucidate if the changes occur immediately after transmission to a new host.

We were not able to show any Lewis antigen switching in our *in vitro* experiments of *H. pylori* isolates grown at different pH, as described by Moran *et al.* [25] (Figure 1). Thus, our isolates demonstrated the same Lewis antigen expression at both pH 5 and pH 7. However, three of the five wildtype mouse isolates presented O-chains with reduced levels of Lewis antigens when grown at pH 5 as compared to pH 7 (Figure 1). The pH differences in Lewis antigen expression were much less apparent in the original challenge strain and in the four isolates obtained from the ChAG mouse model. Hence, unknown genetic changes affecting Lewis antigen expression levels may be selected in different mouse gastric environments. pH-dependent effects on Lewis antigen expression probably depend, at least partially, on a reduced production of high- and low-molecular weight structures in the LPS profiles of the isolates grown at pH 5 as compared to pH 7, suggesting that growth at low pH provides less substrate available for fucosylation.

In the human setting, it is less clear how gastric atrophy with reduced acid-output affect the pH at the epithelial layer where *H. pylori* is replicating. Clinical isolates from normal individuals produced less LPS structures after *in vitro* growth at pH 5 as compared to pH 7. This pH regulation of LPS expression was much less apparent for most of the atrophy-associated isolates tested. Among the atrophy-associated isolates there were no differences in pH response for isolates obtained at year 0 as compared to isolates taken from the same stomach four years later, except for two cases (Kx438 and Kx1039). Since our comparative genetics data suggest that isolates within single individuals belong to the same strain, it appears that a regulatory shift in pH regulation of LPS expression occurred simultaneously with the shift from atrophy to gastric adenocarcinoma or from mild atrophy to high grade atrophy. It is clear however, that we need to identify more such cases before we can suggest causal relationships.

Phenotypic variation of surface-exposed structures is a means for bacteria to adapt to varying environmental conditions and to resist the wide range of immune responses of the host. *H. pylori*, *Campylobacter jejuni*, *Neisseria meningitidis*, *Haemophilus influenzae* and *Salmonella* spp. etc. are able to modify LPS carbohydrate epitopes through transcriptional regulation of glycosyltransferases [10], [38], [39]. *H. pylori* LPS is highly diverse due to genetic changes and transcriptional regulation of the enzymes that are involved in LPS biosynthesis and glycosylation. *In vitro* studies have shown that the expression of Lewis antigens can phase vary at high frequencies (0.2–0.5%), which favors strain diversification and suggest that several different LPS phenotypes can exist in a bacterial population originating from a single cell [8]. The variation in fucosylation can be a result of phase variation through slipped-strand mispairing due to varying numbers of polyC repeats in the fucosyltransferase genes *futA*, *futB* and *futC*. In addition, Lewis expression can vary depending on the relative activities of the α1,2 and α1,3-fucosyltransferases as well as on fucose availability [21]. The *H. pylori wbcJ* gene, homologous to O-antigen chain biosynthesis genes involved in the conversion of GDP-D-mannose to GDP-D-fucose, has been shown to be acid-induced by subtractive RNA hybridization [40]. Thus the notion that fucose availability is dependent on the environmental pH suggests that fucose levels can vary in different gastric compartments depending on the parietal-cell content and that the levels can be altered in response to gastric disease progression since the gastric pH increases when parietal cells are destroyed.

*H. pylori* is an extremely diverse species and since phase variation of fucosyltransferases occurs at high frequencies, bacteria can present LPS with different glycosylation patterns. We have shown that different LPS variants exist within individuals during their lifetime, which depends on the gastric environment. Thus, maintaining LPS diversity within the sub-population is important in regulating the host's inflammatory response and to adapt to the changing gastric environment during disease progression.

## Materials and Methods

### Bacterial strains

Lewis antigen expression was investigated in HPAG1 single-colony re-isolates after a one-year colonization of parietal cell-deficient mice (*tox*176 mice; five single-colony re-isolates from two mice), and non-transgenic FVB/N mice (wildtype mice; five single-colony re-isolates from four mice) [27]. All manipulations involving mice were performed by using protocols approved by the Washington University Animal Studies Committee. Strain HPAG1 [41] was initially obtained from an individual with ChAG in a Swedish case-control study of gastric cancer [41], [42], which was approved by the Ethics Committee of Uppsala University (Uppsala, Sweden 1995; written informed consent was obtained from all participants). Furthermore, 482 clinical *H. pylori* isolates obtained from corpus biopsies from 17 selected individuals (ages 52–75) included in a population-based upper-endoscopy study, the Kalixanda study, at an initial and a follow-up endoscopy four years apart [43], [44], were studied to determine Lewis antigen phenotypes and diversity. During this period, six individuals progressed from low grade atrophy to severe atrophy or gastric adenocarcinoma, two individuals with initially normal mucosa established atrophy and nine individuals had normal mucosa at both occasions, as indicated by both histology and serum pepsinogen levels. Fifteen single-colony isolates were obtained from each individual and time point (except individuals Kx1167 and Kx1353 where only three and 14 isolates, respectively, could be obtained from year 0, and Kx439 where no isolates could be obtained from year 4). The Kalixanda study was approved by the Ethics Committee of Umeå University (Umeå, Sweden, 1998) and written informed consent was obtained from all participants [43], [44]. Clinical *H. pylori* isolates from the Kalixanda study that were used in this study are listed in Table 2.

### Culture conditions

Bacteria were grown on GC agar plates at 37°C under microaerophilic conditions, as previously described [29]. For LPS isolation, bacteria were cultured in Brucella broth (Becton Dickinson, Cockeysville, MD) supplemented with 5% FBS (Sigma, St Louis, MO) and 1% IsoVitaleX (Becton Dickinson), pH 7.0, to exponential phase. For growth at different pH, cultures were divided at mid-log phase (OD_600_≈0.5) and one half was exposed to Brucella broth with pH 5, while the other half was maintained at pH 7. Bacteria were harvested after 24 h followed by subsequent LPS isolation.

### LPS isolation

Equal amounts of bacteria (as determined by OD_600_) were collected from the liquid cultures. Samples were harvested by centrifugation and washed twice in PBS. LPS was isolated using the hot phenol-water technique, as previously described [22]. LPS was extracted twice with incubation in equal volumes of water and phenol for 15 min at 70°C with repeated vortexing. After centrifugation at 16,000×*g* for 15 min at 4°C, the aqueous phases were pooled and precipitated overnight at −20°C in 10 volumes of 99.5% ethanol and sodium acetate (final concentration 0.03 M). LPS was precipitated by centrifugation (16,000×*g* for 20 min, 4°C), washed in 70% ethanol, air-dried, and then resuspended in water.

### Polyacrylamide Gel Electrophoresis and Immunoblotting

LPS preparations were separated by SDS-PAGE using a 4% polyacrylamide stacking gel and a 15% polyacrylamide separating gel. The gels were stained with silver or blotted to PVDF membranes (BioRad, Hercules, CA) for immunodetection. Membranes were blocked overnight in blocking buffer [PBS supplemented with 1% bovine serum albumin, (BSA; Sigma) and 0.1% Tween-20] at 4°C. Thereafter, membranes were incubated with the primary antibodies, mouse anti-Le^x^ (MCA1762 or MCA1313, both from Serotec, Oxford, UK) diluted 1∶400, or mouse anti-Le^y^ (MCA1091, Signet Laboratories, Dedhan, MA) diluted 1∶4,000, in blocking buffer for 1 h at room temperature. After washes in PBS-T [PBS supplemented with 0.1% Tween-20], membranes were incubated with the secondary antibody, horseradish peroxidase-conjugated goat anti-mouse antibody (STAR86, Serotec), diluted 1∶1,000 in blocking buffer for 1 h at room temperature, and finally washed in PBS-T. Membranes were developed with enhanced chemiluminescence (ECL; Amersham Pharmacia Biosciences, Buckinghamshire, UK) and then exposed to Hyperfilm ECL (Amersham Pharmacia Biosciences) for chemiluminescence detection.

### Genetic analyses

*H. pylori* DNA was isolated using a DNeasy® Tissue Kit (Qiagen; Hilden, Germany) or Amplicor Respiratory Specimen Preparation Kit (Roche Diagnostics GmbH, Mannheim, Germany) according to the manufacturer's protocol. Genomic fingerprinting, using randomly amplified polymorphic DNA PCR (RAPD-PCR) with DyNAzyme *Taq* Polymerase and the corresponding buffers (Finnzymes, Espoo, Finland), was performed on the clinical isolates. PCR and DNA-sequencing of *cagA*, *cag* PAI empty site, *futA* and *futB* were performed using primers listed in Table S1. PCR was performed under standard conditions with Invitrogen Taq DNA polymerase and the corresponding buffers (Invitrogen, Carlsbad, CA). Cycle-sequencing reactions were performed using a BigDye® Terminator v.3.1 Cycle Sequencing Kit (Applied Biosystems, Foster City, CA) and samples were subsequently analyzed on ABI PRISM® 3100 Genetic Analyzer (Applied Biosystems). DNA sequences were assembled and aligned using the Vector NTI 10.3.0 software package (Invitrogen).

### Serum analysis

Serum pepsinogen I and II (PGI and PGII) concentrations of serum collected at the first endoscopy from individuals in the Kalixanda study were measured by enzyme immunoassay (EIA; Biohit Plc, Helsinki, Finland) according to the manufacturer's instructions.

## Supporting Information

Table S1 Primers used in this study.
(0.03 MB PDF)

Figure S1 Lewis antigen expression in clinical *H. pylori* isolates shows large intra-individual diversity. Fifteen single-colony isolates from each individual and time point were obtained. Immunoblot analysis with antibodies detecting Le^x^ and Le^y^ antigens showed considerable intra-strain diversity of Lewis epitopes within individuals, however the Lewis antigen expression was stable over the four-year period in both normal as well as in atrophic individuals. Lewis antigen expression levels, pattern of Lewis antigen glycosylation and the sizes of O-antigen chains that were fucosylated, also varied among isolates obtained from the same individual. The most common LPS phenotype was Le^y^, either alone, or in combination with Le^x^, whereas the least common was Le^x^ exclusively.
(10.35 MB PDF)

Figure S2 Lewis phenotypes of clinical *H. pylori* isolates grown *in vitro* at pH 7 and pH 5. LPS was extracted from one single-colony isolate from six individuals (Kx345, Kx438, Kx1039, Kx1172, Kx1259 and Kx1379) at both time points. Immunoblot analysis detecting Le^x^ and Le^y^ showed that levels of Lewis antigen expression differed slightly at pH 7 and pH 5, e.g. in isolates Kx345:4, Kx438:0 and Kx1379:4, which showed reduced expression of Le^y^ at pH 5 as compared to pH 7.
(0.72 MB TIF)
