# Supplementary material for: A Changing Gastric Environment Leads to Adaptation of Lipopolysaccharide Variants in Helicobacter pylori Populations during Colonization
Source: PLoS One. 2009 Jun 11;4(6):e5885. doi: 10.1371/journal.pone.0005885 (PMC2690825; doi:10.1371/journal.pone.0005885)
Supplement: Table S1 — Primers used in this study. (0.03 MB PDF) [file pone.0005885.s001.pdf]

Table S1. Primers used in this study.

Primer name	Sequence 5'-3'	Target gene(s)	Application	Reference
HP0379 F	CTC TCG TGA TCT TGG CTT ATT	*futA* upstream	Seq	[1]
HP0379 R	AAG TAG CGT CTG CGA TGA	*futA* downstream	Seq	[1]
C-tract up 651	GCC CTA ATC AAG CCT TTG	*futB* upstream	Seq	[1]
Repeat down 651	AAA ACC CCA CGC TCA AAA A	*futB* downstream	Seq	[1]
FucT F1	TTC CAA CCC CTA TTA GAC G	*futA*, *futB*	PCR, Seq	[2]
FucT F3	GAT CGT TAT TTG AGA ATG CC	*futA*, *futB*	Seq	[2]
FucT F4	CAA AGA CAA CAG CCT TTA TGC	*futA*, *futB*	Seq	[2]
FucT F5	GCG AGT TTT TAA GCC AAT AC	*futA*, *futB*	Seq	[2]
FucT R1	GTT AAA ATC TTT CGC CAC G	*futA*, *futB*	PCR, Seq	[2]
FucT R2	GGG TGT TTA AAG GGT TTT C	*futA*, *futB*	Seq	[2]
FucT R3	TTT CTC ACA CTT CCT CCC C	*futA*, *futB*	Seq	[2]
FucT R4	GTT GCT CGC TAC AAA ACT G	*futA*, *futB*	Seq	[2]
cagAsbra F	ATG ATG GCG TGA TGT TTG T	*cagA*	PCR	[3]
cagAsbra R	TTT TCA AGG TCG CTT TTT GC	*cagA*	PCR	[3]
UpCagF	ACT TTC ACG CCC TTT CCC TCC	*cag* PAI empty site	PCR	[3]
DownCagR	TTG CAT GCG TTA TTA TTT CAC	*cag* PAI empty site	PCR	[3]
1290	GTG GAT GCG A	randomly	RADP	[4]
1283	GCG ATC CCC A	randomly	RADP	[4]

1. Nilsson C, Skoglund A, Moran AP, Annuk H, Engstrand L, et al. (2006) An enzymatic ruler modulates Lewis antigen glycosylation of Helicobacter pylori LPS during persistent infection. Proc Natl Acad Sci U S A 103: 2863-2868.
2. Nilsson C, Skoglund A, Moran AP, Annuk H, Engstrand L, et al. (2008) Lipopolysaccharide diversity evolving in Helicobacter pylori communities through genetic modifications in fucosyltransferases. PLoS ONE 3: e3811.
3. Nilsson C, Sillen A, Eriksson L, Strand ML, Enroth H, et al. (2003) Correlation between cag pathogenicity island composition and Helicobacter pylori-associated gastroduodenal disease. Infect Immun 71: 6573-6581.
4. Akopyanz N, Bukanov NO, Westblom TU, Berg DE (1992) PCR-based RFLP analysis of DNA sequence diversity in the gastric pathogen Helicobacter pylori. Nucleic Acids Res 20: 6221-6225.
