# Supplementary material for: A Changing Gastric Environment Leads to Adaptation of Lipopolysaccharide Variants in Helicobacter pylori Populations during Colonization
Source: PLoS One. 2009 Jun 11;4(6):e5885. doi: 10.1371/journal.pone.0005885 (PMC2690825; doi:10.1371/journal.pone.0005885)
Supplement: Figure S1 — Lewis antigen expression in clinical H. pylori isolates shows large intra-individual diversity. Fifteen single-colony isolates from each individual and time point were obtained. Immunoblot analysis with antibodies detecting Lex and Ley antigens showed considerable intra-strain diversity of Lewis epitopes within individuals, however the Lewis antigen expression was stable over the four-year period in both normal as well as in atrophic individuals. Lewis antigen expression levels, pattern of Lewis antigen glycosylation and the sizes of O-antigen chains that were fucosylated, also varied among isolates obtained from the same individual. The most common LPS phenotype was Ley, either alone, or in combination with Lex, whereas the least common was Lex exclusively. (10.35 MB PDF) [file pone.0005885.s002.pdf]

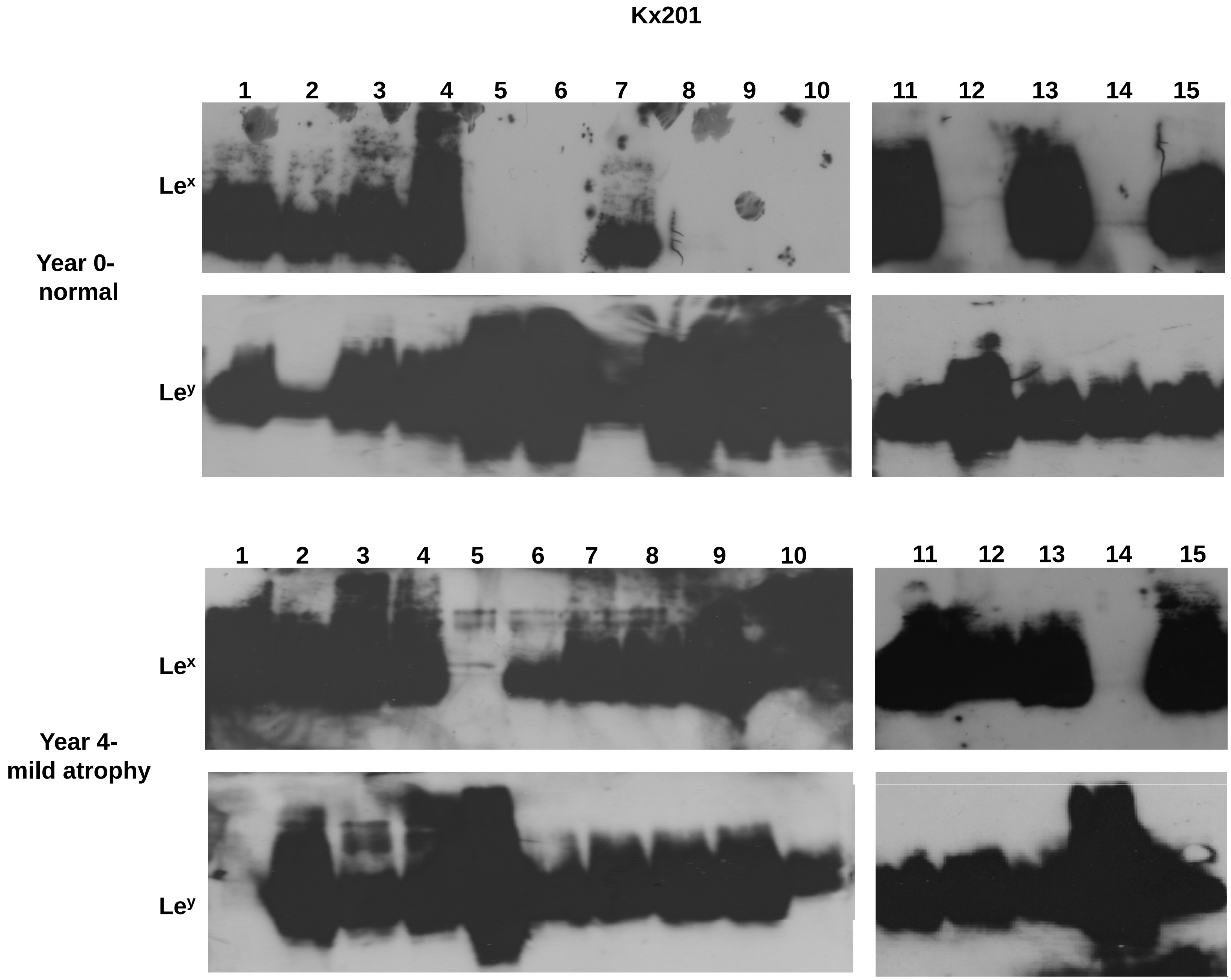

Kx201
1 2 3 4 5 6 7 8 9 10 11 12 13 14 15
Le^{x}
Le^{y}
Year 0-
normal
1 2 3 4 5 6 7 8 9 10 11 12 13 14 15
Le^{x}
Le^{y}
Year 4-
mild atrophy

Kx208

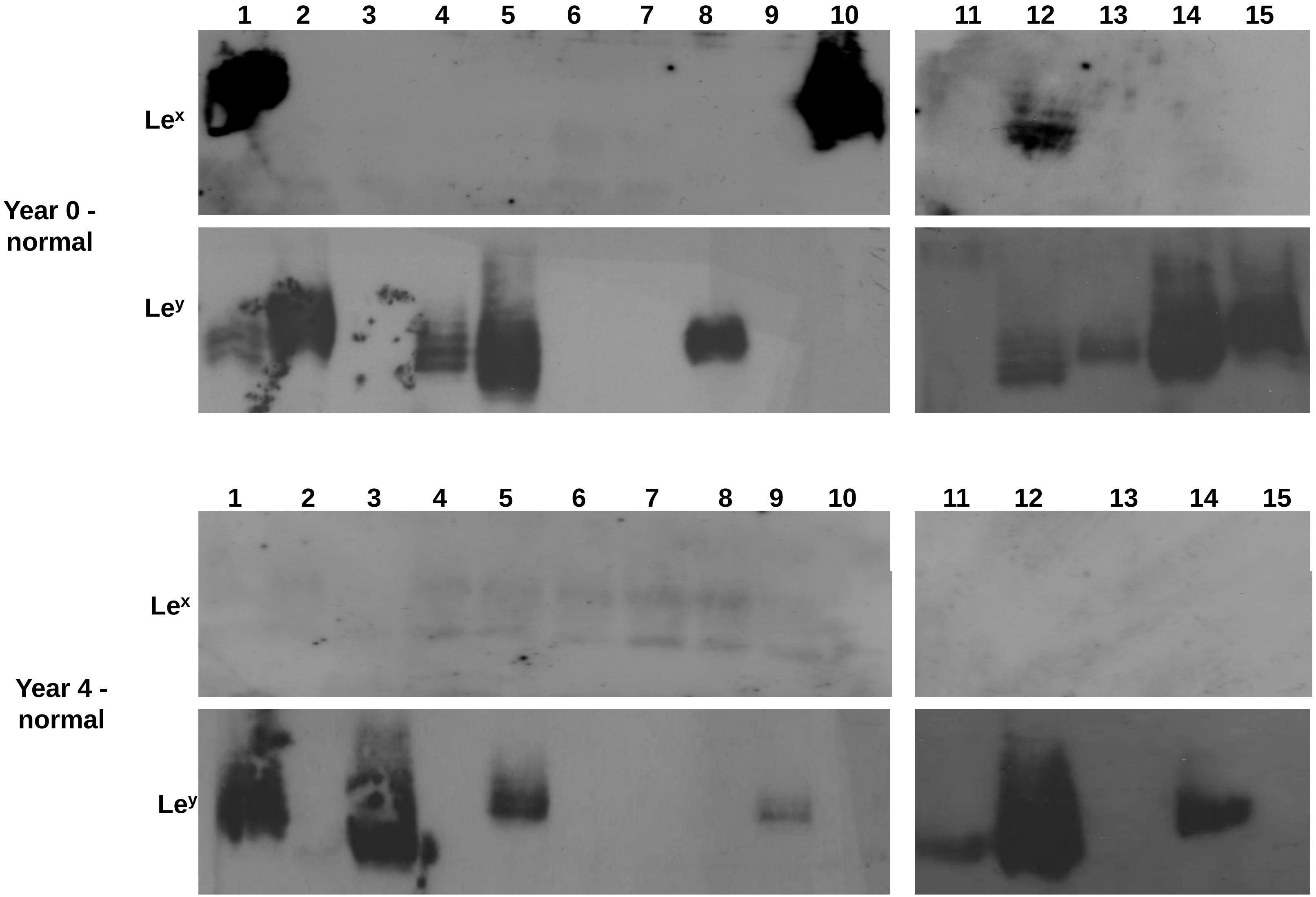
1 2 3 4 5 6 7 8 9 10
11 12 13 14 15
Le^{x}
Le^{y}
Year 0 - normal
1 2 3 4 5 6 7 8 9 10
11 12 13 14 15
Le^{x}
Le^{y}
Year 4 - normal

Kx239

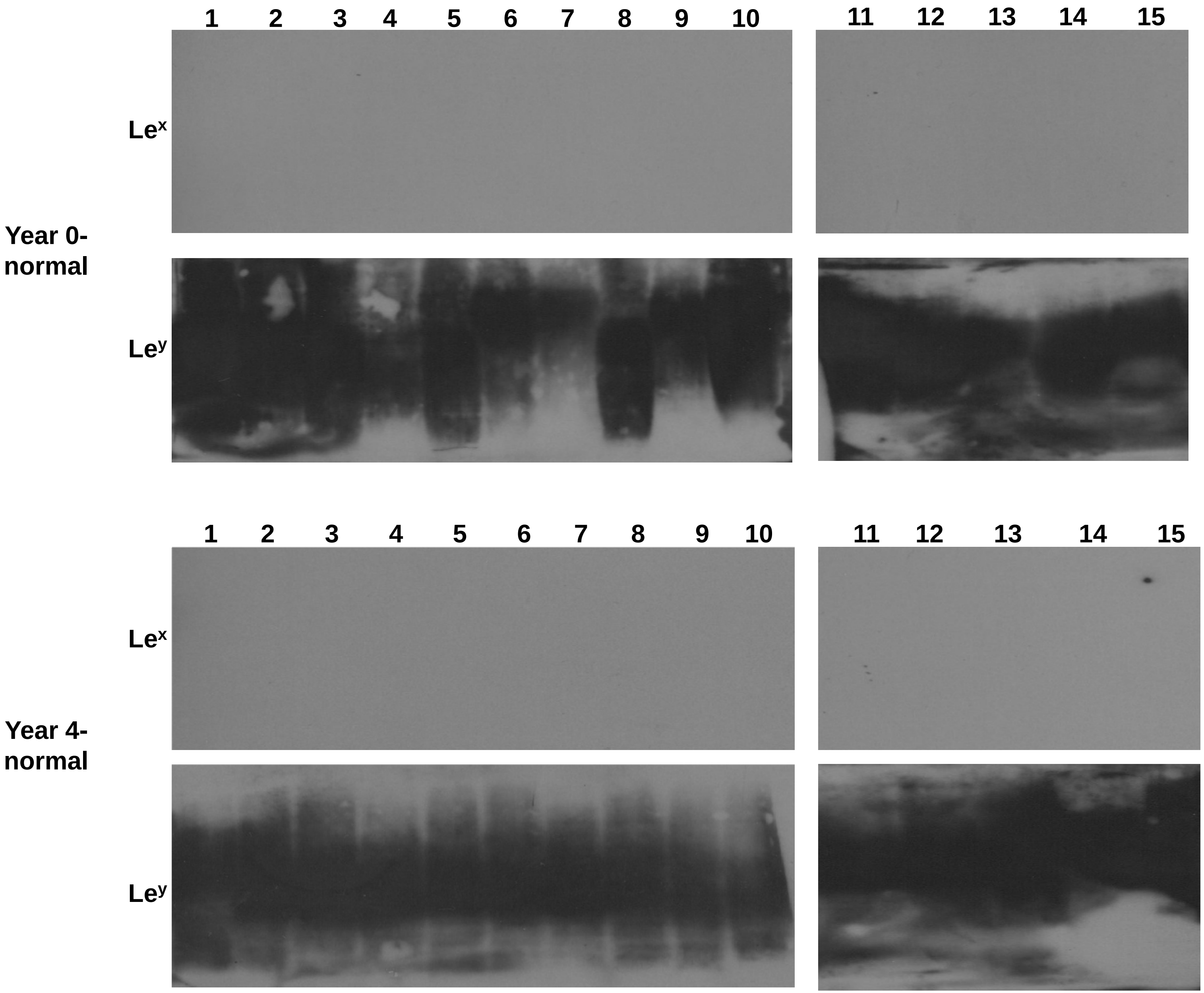

1 2 3 4 5 6 7 8 9 10
11 12 13 14 15
Le^{x}
Year 0-
normal
Le^{y}
1 2 3 4 5 6 7 8 9 10
11 12 13 14 15
Le^{x}
Year 4-
normal
Le^{y}

Kx364

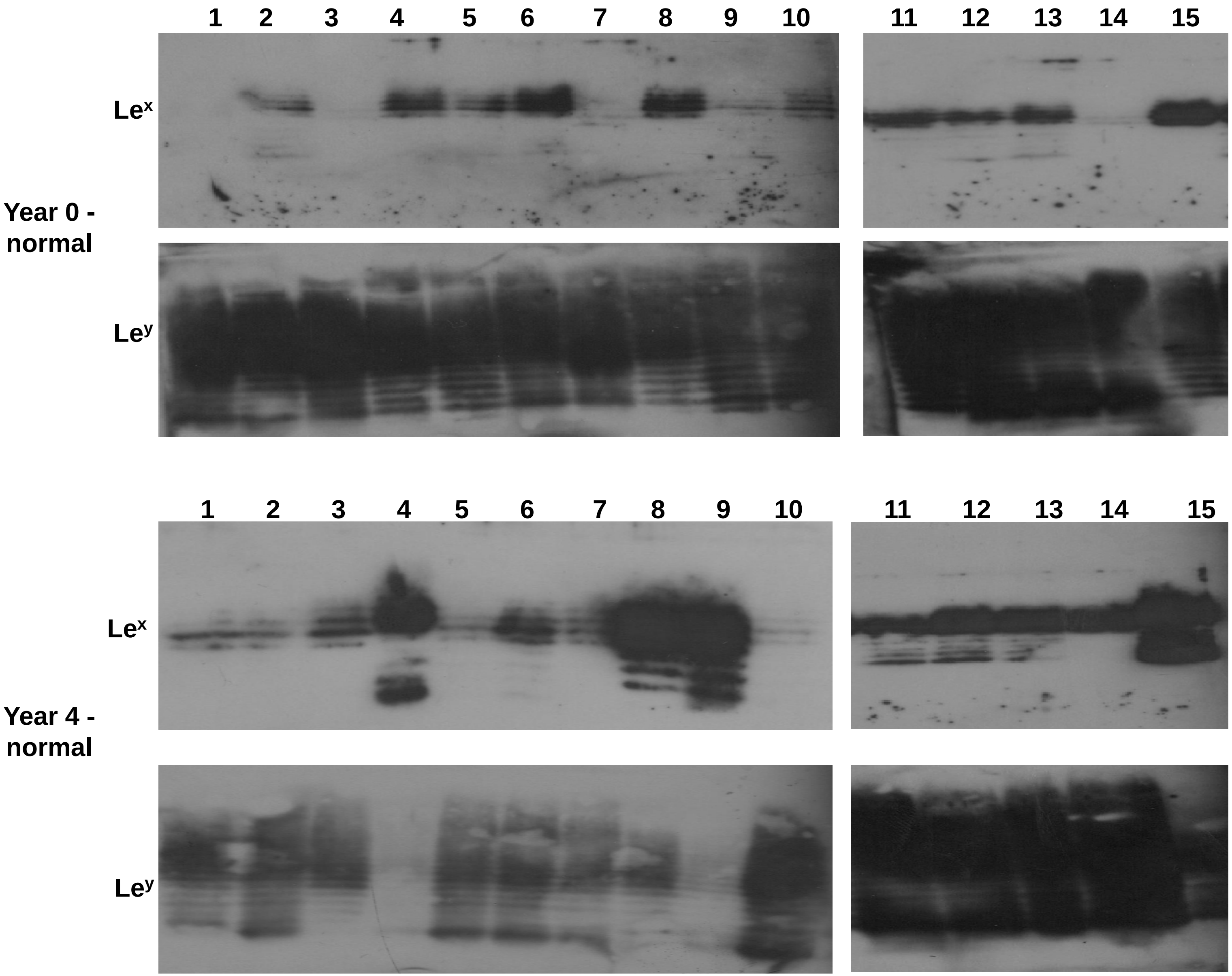
1 2 3 4 5 6 7 8 9 10
11 12 13 14 15
Le^x
Le^y
Year 0 - normal
1 2 3 4 5 6 7 8 9 10
11 12 13 14 15
Le^x
Le^y
Year 4 - normal

Kx439

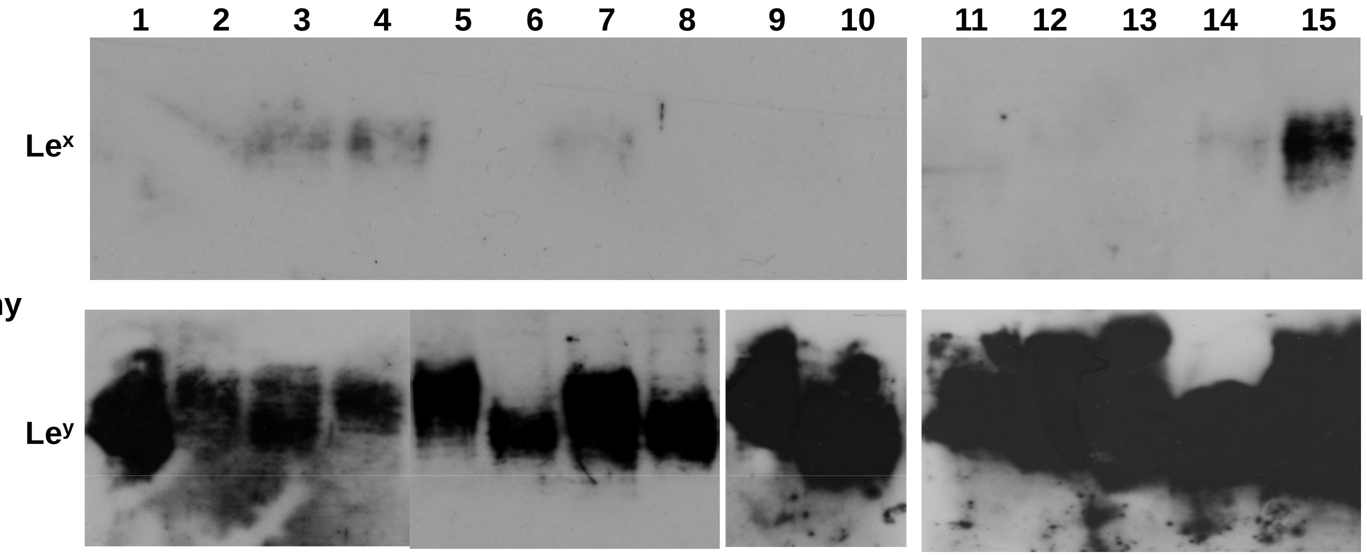

Year 0 -
mild atrophy

Year 4 -
high grade atrophy

No isolates available from year 4

Kx491

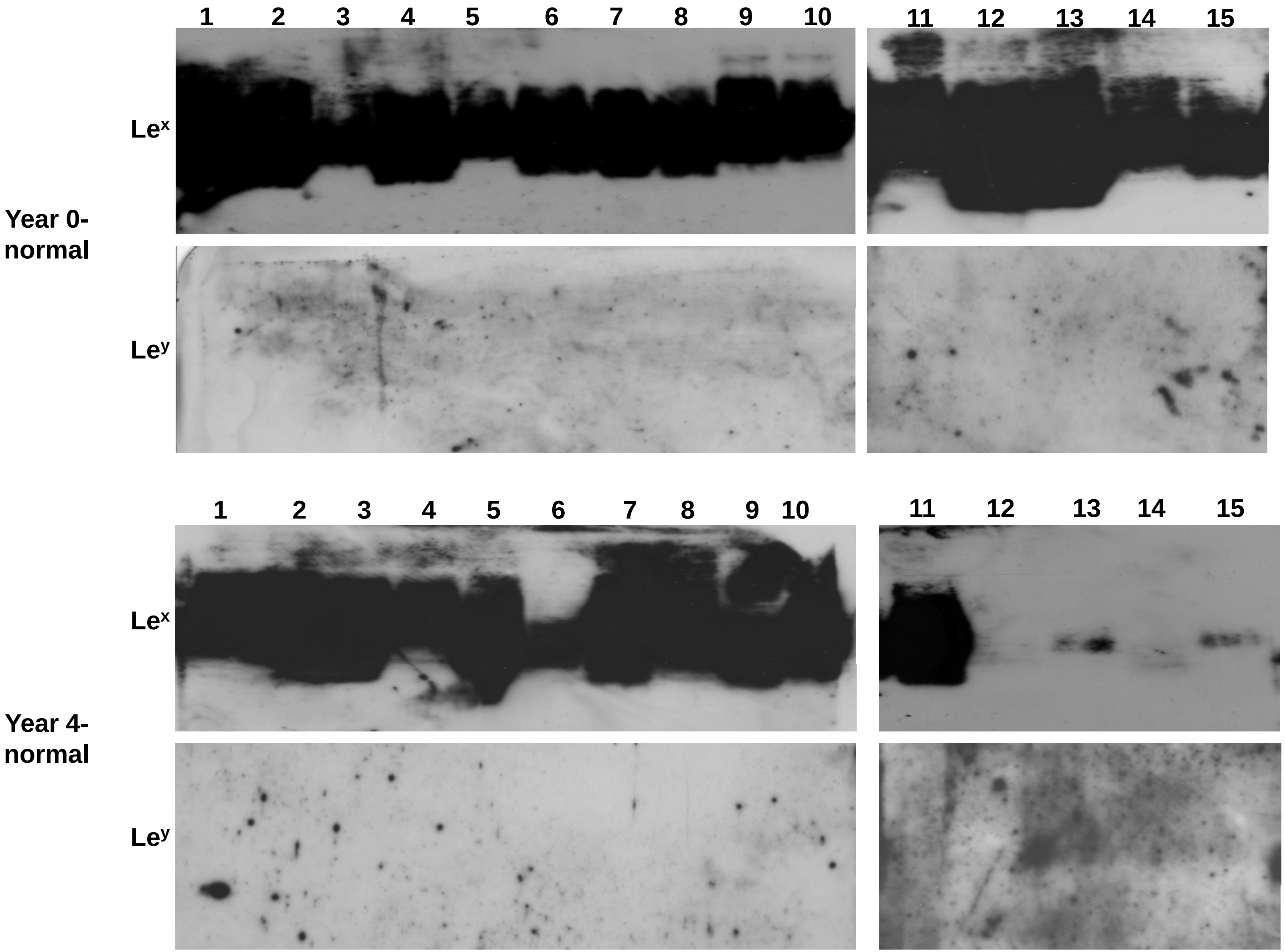
1 2 3 4 5 6 7 8 9 10
11 12 13 14 15
Le^x
Le^y
Year 0- normal
1 2 3 4 5 6 7 8 9 10
11 12 13 14 15
Le^x
Le^y
Year 4- normal

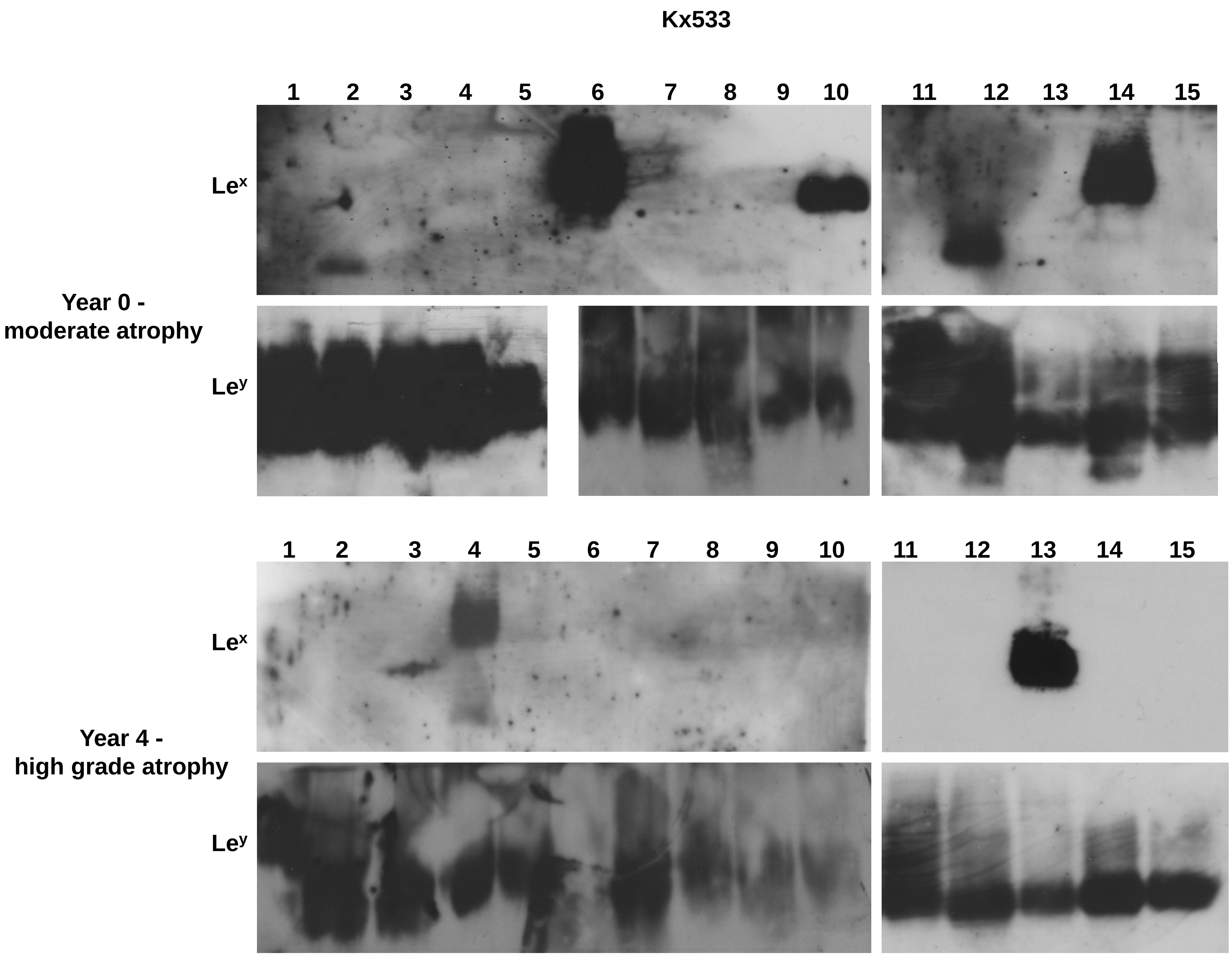
Kx533
1 2 3 4 5 6 7 8 9 10 11 12 13 14 15
Le^x
Le^y
Year 0 -
moderate atrophy
1 2 3 4 5 6 7 8 9 10 11 12 13 14 15
Le^x
Le^y
Year 4 -
high grade atrophy

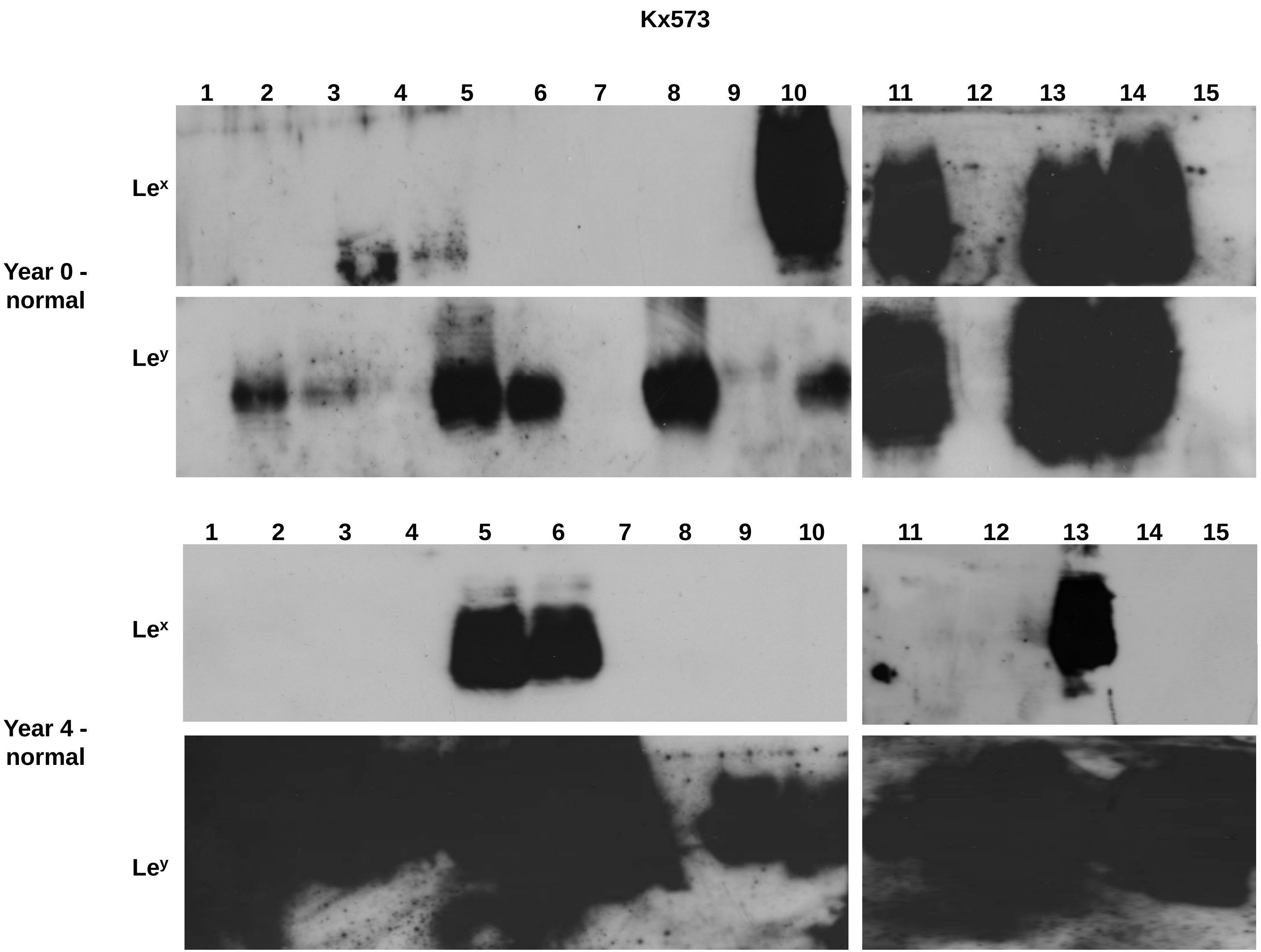
Kx573
1 2 3 4 5 6 7 8 9 10 11 12 13 14 15
Le^x
Le^y
Year 0 - normal
1 2 3 4 5 6 7 8 9 10 11 12 13 14 15
Le^x
Le^y
Year 4 - normal

Kx595

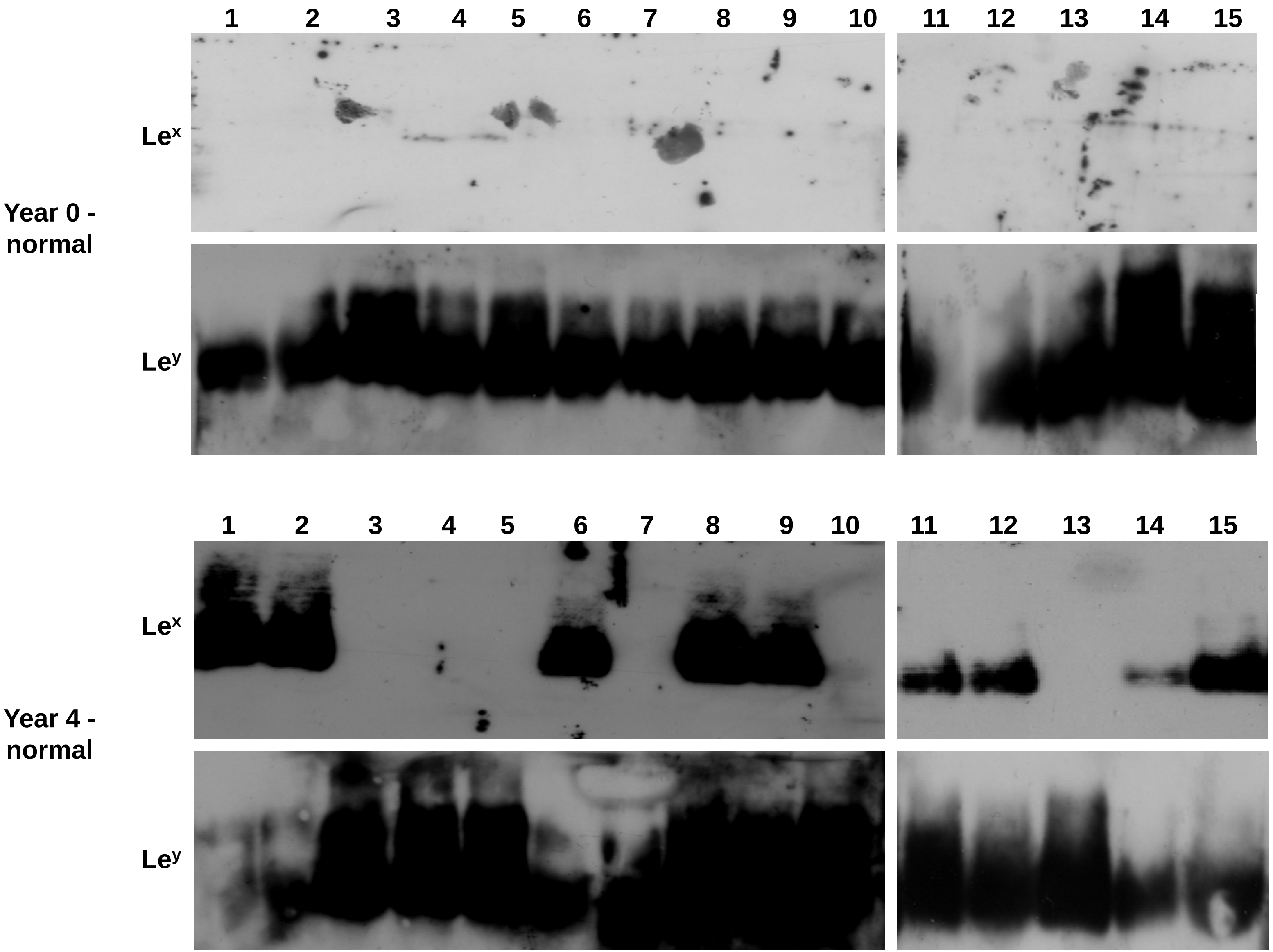
1 2 3 4 5 6 7 8 9 10 11 12 13 14 15
Le^x
Le^y
Year 0 - normal
1 2 3 4 5 6 7 8 9 10 11 12 13 14 15
Le^x
Le^y
Year 4 - normal

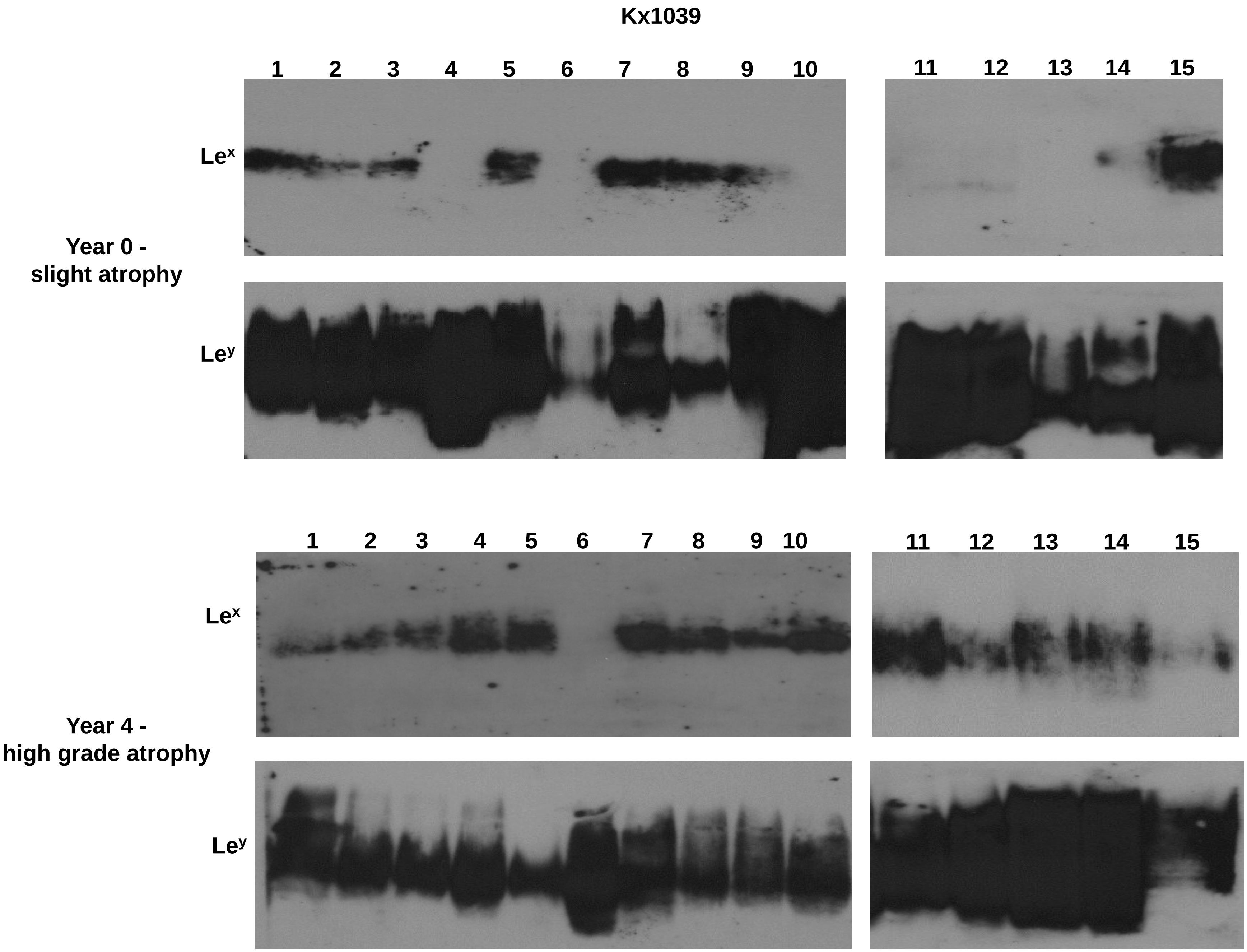
Kx1039
1 2 3 4 5 6 7 8 9 10
11 12 13 14 15
Le^x
Le^y
Year 0 -
slight atrophy
1 2 3 4 5 6 7 8 9 10
11 12 13 14 15
Le^x
Le^y
Year 4 -
high grade atrophy

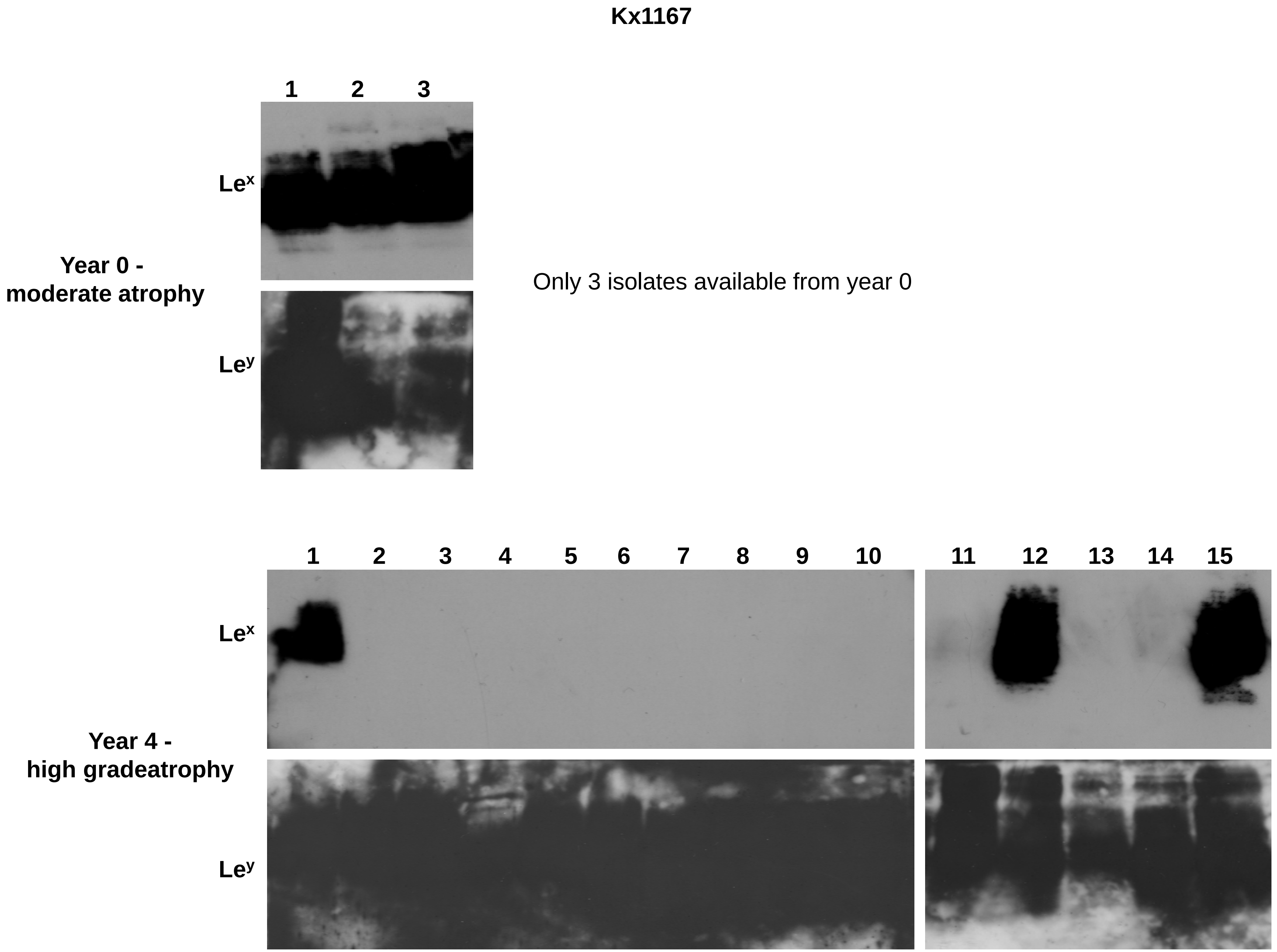
Kx1167
1 2 3
Le^x
Le^y
Year 0 -
moderate atrophy
Only 3 isolates available from year 0
1 2 3 4 5 6 7 8 9 10 11 12 13 14 15
Le^x
Le^y
Year 4 -
high gradeatrophy

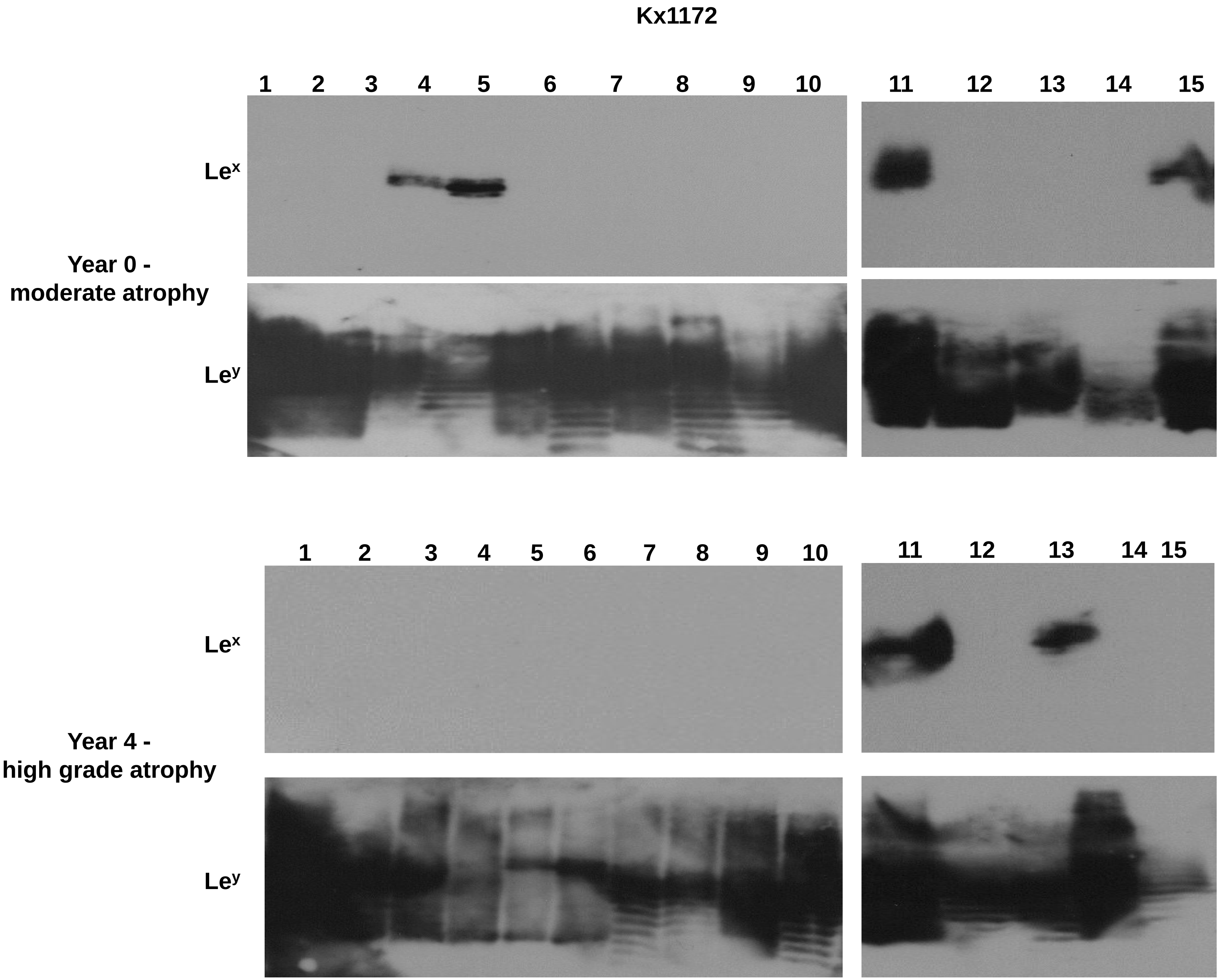
Kx1172
1 2 3 4 5 6 7 8 9 10
11 12 13 14 15
Le^x
Le^y
Year 0 -
moderate atrophy
1 2 3 4 5 6 7 8 9 10
11 12 13 14 15
Le^x
Le^y
Year 4 -
high grade atrophy

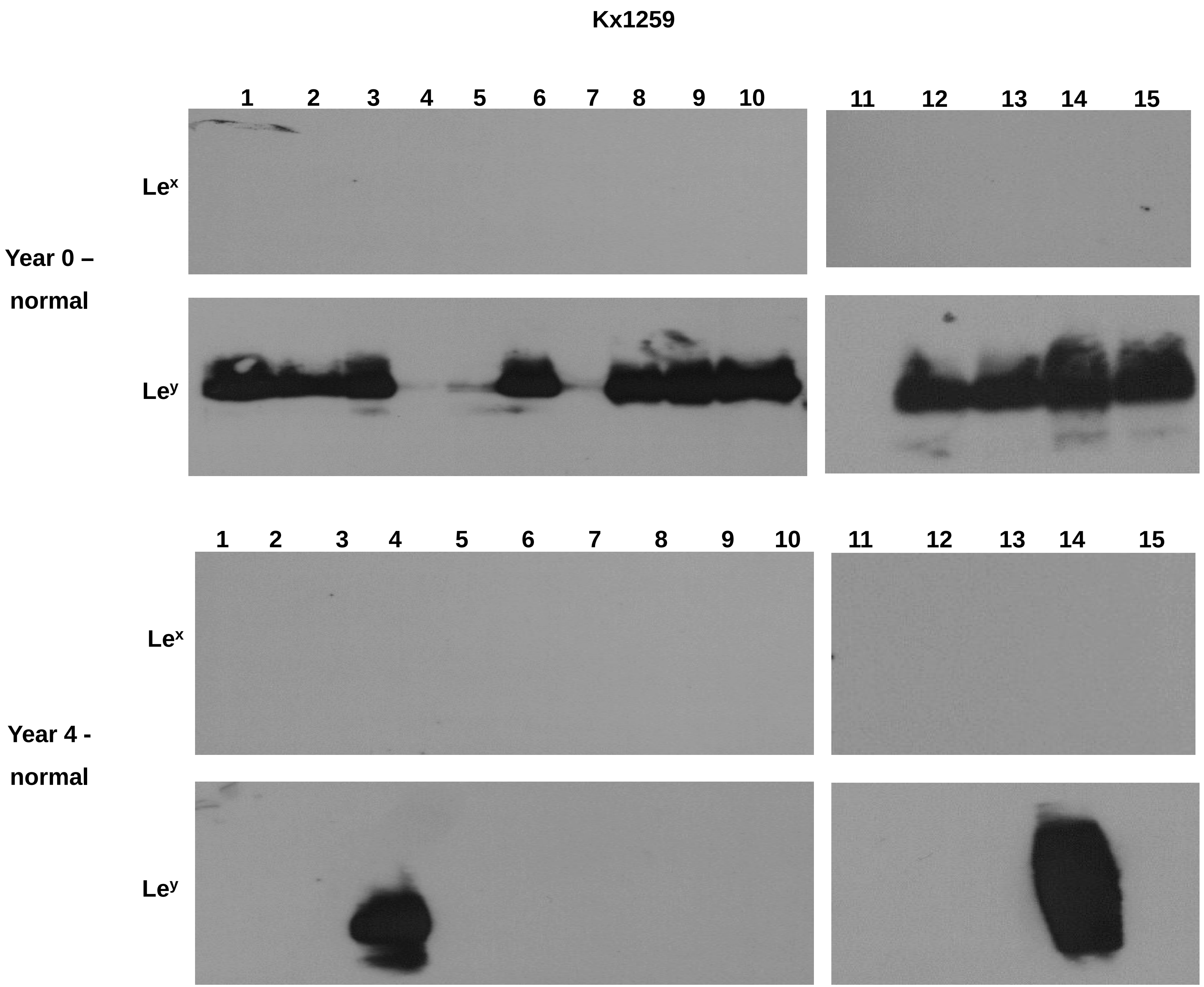
Kx1259
1 2 3 4 5 6 7 8 9 10
11 12 13 14 15
Le^{x}
Le^{y}
Year 0 –
normal
1 2 3 4 5 6 7 8 9 10
11 12 13 14 15
Le^{x}
Le^{y}
Year 4 -
normal

Kx1353

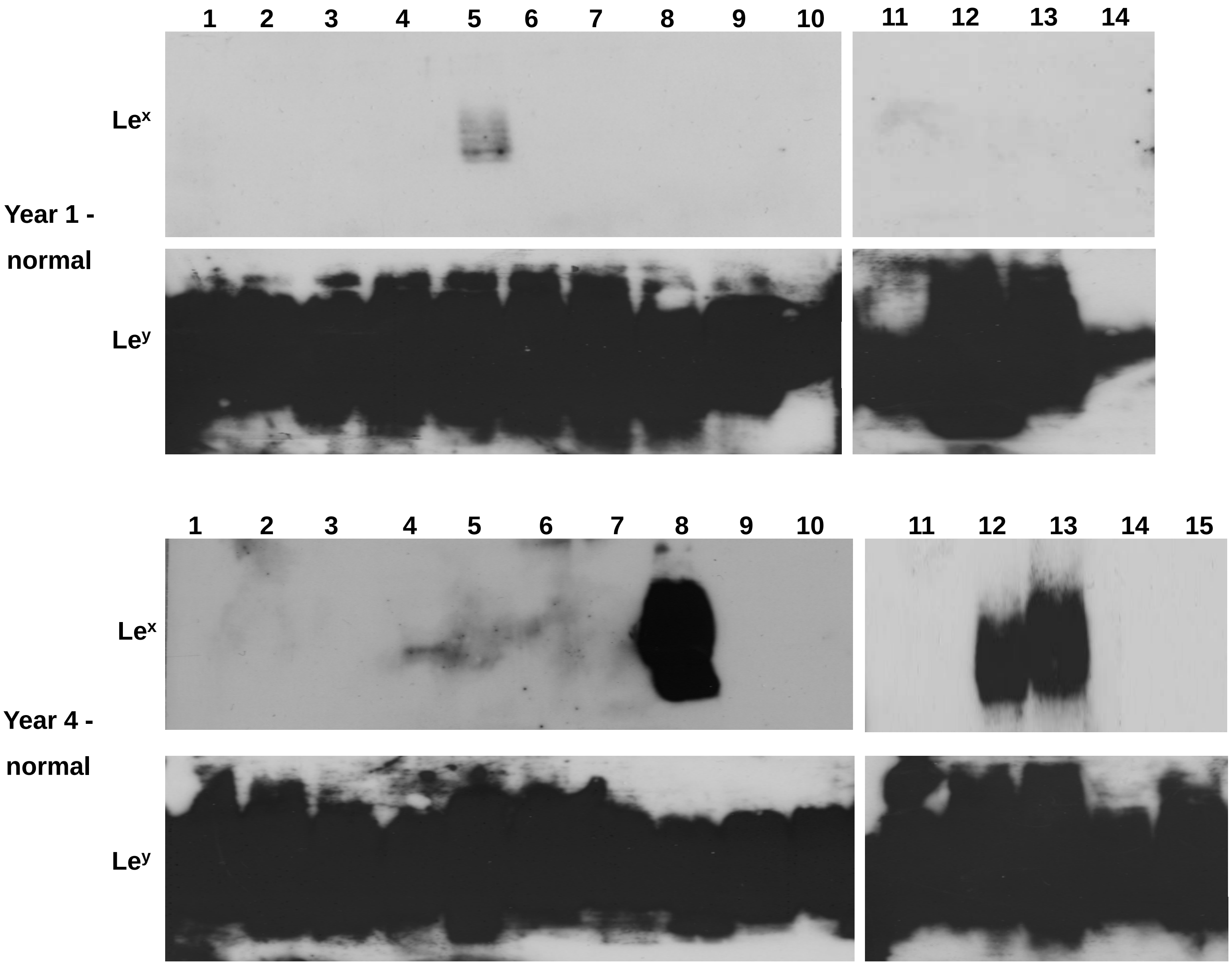
1 2 3 4 5 6 7 8 9 10 11 12 13 14
Le^x
Year 1 -
normal
Le^y
1 2 3 4 5 6 7 8 9 10 11 12 13 14 15
Le^x
Year 4 -
normal
Le^y

Fig. S1. Lewis antigen expression in clinical *H. pylori* isolates shows large intra-individual diversity.

Fifteen single-colony isolates from each individual and time point were obtained. Immunoblot analysis with antibodies detecting $Le^x$and Le^y antigens showed considerable intra-strain diversity of Lewis epitopes within individuals, however the Lewis antigen expression was stable over the four-year period in both normal as well as in atrophic individuals. Lewis antigen expression levels, pattern of Lewis antigen glycosylation and the sizes of O-antigen chains that were fucosylated, also varied among isolates obtained from the same individual. The most common LPS phenotype was Le^y, either alone, or in combination with Le^x, whereas the least common was Le^x exclusively.
