# Supplementary figures and images for: A Changing Gastric Environment Leads to Adaptation of Lipopolysaccharide Variants in Helicobacter pylori Populations during Colonization
Source: PLoS One. 2009 Jun 11;4(6):e5885. doi: 10.1371/journal.pone.0005885 (PMC2690825; doi:10.1371/journal.pone.0005885)

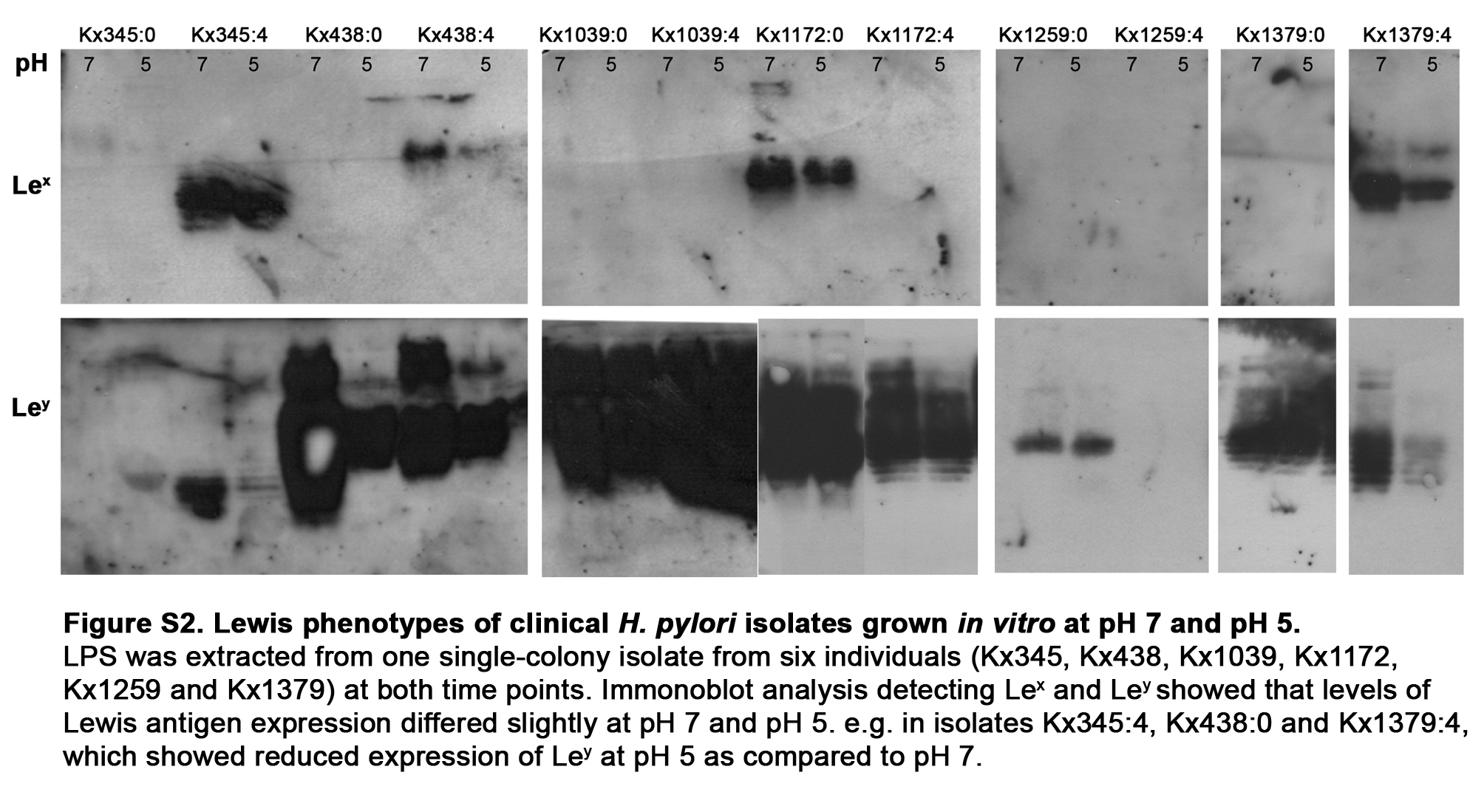

Supplement: Figure S2 — Lewis phenotypes of clinical H. pylori isolates grown in vitro at pH 7 and pH 5. LPS was extracted from one single-colony isolate from six individuals (Kx345, Kx438, Kx1039, Kx1172, Kx1259 and Kx1379) at both time points. Immunoblot analysis detecting Lex and Ley showed that levels of Lewis antigen expression differed slightly at pH 7 and pH 5, e.g. in isolates Kx345:4, Kx438:0 and Kx1379:4, which showed reduced expression of Ley at pH 5 as compared to pH 7. (0.72 MB TIF) [file pone.0005885.s003.tif]
